# Involving Patients in Pharmacoepidemiology Studies Conducted on Healthcare Administrative Database: Results of a Feasibility Study in France

**DOI:** 10.1111/hex.70879

**Published:** 2026-09-21

**Authors:** Julie Rigoureau, Manon Verroul, Valentine Vieillard, Anne Termoz, Laurie Panse, Anaïs Havet, Marie Viprey, Julie Haesebaert

**Affiliations:** ^1^ Research on Healthcare Performance (RESHAPE), INSERM U1290 Université Claude Bernard Lyon 1 Lyon France; ^2^ Hospices Civils de Lyon Service de Recherche et épidémiologie cliniques Lyon France; ^3^ Hospices Civils de Lyon Service Données de Santé Lyon France

**Keywords:** feasibility study, patient and public involvement, pharmacoepidemiology

## Abstract

**Background:**

Patient and public involvement in pharmacoepidemiology using healthcare administrative databases remains limited despite its potential to enhance research relevance and societal impact. We evaluated the feasibility and benefits of involving patients as research partners in a Patient Committee for a pharmacoepidemiological study using the French nationwide health insurance database (*SNDS*).

**Methods:**

A participatory feasibility study was conducted through the establishment of a Patient Committee alongside the study's Scientific Committee. A mixed‐methods evaluation assessed feasibility combining quantitative indicators, analysis of group dynamics, and thematic analysis of the transcription of the second meeting. Benefits were evaluated based on changes made in protocol, statistical analysis, and civil society dissemination plans based on patients' proposals. The McMaster tool was used to assess the involvement process.

**Results:**

Three patients were recruited out of the minimum expected five. Of the five meetings, three were attended by all participants. Consensus was reached on most points discussed and a good cohesion was observed within the Committee. The questions asked to the moderators focused primarily on the progress of the research and on the availability of data beyond the reimbursement‐related data available in the SNDS, as data related to the patient's perspective (quality of life or anxiety). At the second meeting, only 13% of coded exchanges fell within the categories previously predefined according to the meeting's objectives. The remaining themes were unrelated to their illness, drug utilisation or care pathways. Patient involvement led to an effective modification of a primary outcome in the research protocol and an additional statistical analysis. Patient Committee also contributed several points for discussion and produced two visual abstracts. Finally, patients expressed satisfaction with their participation, feeling that their opinions had been listened to and taken into account. Project team members involved in project management appeared satisfied with the process, except in logistical needs (very long research timeline with specific deadlines to be met).

**Conclusion:**

Although benefits of patient involvement were observed, feasibility was partial due to several obstacles related to participant recruitment and patients' understanding of the *SDNS*. To improve feasibility, future initiatives should co‐construct appropriate methodologies with stakeholders and provide adequate training tailored to the complexity of pharmacoepidemiology research.

**Patient and Public Contribution:**

The study was conducted in collaboration with the patient partner from the research laboratory. She was involved in recruiting the Patient Committee and drafting the recruitment materials (flyer and email). The patient partner also proofread the content of the training session.

## 1 Introduction

Pharmacoepidemiology is a research domain dedicated to the evaluation of real‐life drug utilisation, effectiveness, and safety [1]. Frequently employed to inform public decision‐making regarding the regulation and utilisation of medicines, pharmacoepidemiology typically addresses topics that are closely linked to public health and of significant relevance to civil society [1, 2, 3, 4]. In this context, patient and public involvement, defined as ‘research being carried out ‘with’ or ‘by’ members of the public rather than ‘to’, ‘about’ or ‘for’ them’ [5], might be relevant in pharmacoepidemiology research. Indeed, patients' involvement can improve the relevance of research topics, feasibility of projects, interpretation of results, and communication with civil society [6, 7, 8]. Through the sharing of their experiential knowledge, patients provide insights into drug‐use behaviours, treatment decisions (such as discontinuing medication or switching to alternative treatments), and the consequences of certain prescribing practices on individual care trajectories [9]. In addition, drug effectiveness and drug safety are also branches of pharmacoepidemiology where patient involvement could enhance the interpretation of study outcomes and foster more effective communication of results to civil society [10, 11, 12].

However, pharmacoepidemiological research on secondary data sources like administrative healthcare databases requires complex methodology and specific knowledge of the database used [1]. This technical complexity can pose a significant barrier to patient involvement, especially for individuals without prior exposure to the field of research [13]. To date, most examples of patient involvement have been concentrated in early‐stage drug development or experimental clinical trials [14]. In contrast, there is limited documentation and practical guidance on how to effectively involve patients in database‐based pharmacoepidemiological studies, and none of them directly addressed patient involvement in the development of the research protocol [13, 15, 16].

Encouraged in research [17, 18], patient and public involvement in pharmacoepidemiology still seems underdeveloped. Thus, our study aimed to evaluate the feasibility and the benefit of involving patients as members of the research team in a committee for a pharmacoepidemiological study conducted using the French nationwide health insurance databases (*SNDS*).

## 2 Methods

### 2.1 Study Design

The reporting follows the GRIPP2 (Guidance for Reporting Involvement of Patients and the Public) long form [19], available in Supporting Information: S1.

A participatory feasibility study was conducted using a convergent mixed‐methods [20] evaluation of the involvement process in the PENURIE study. Quantitative data collected through feasibility indicators and a questionnaire administered at the end of the study and the qualitative data derived from content analyses of meeting recordings were collected simultaneously, analysed independently, and integrated at the interpretation stage.

The main objective of the PENURIE study was to assess the effects of cardiovascular and neurologic drug shortages in France between January 2014 and December 2019 on medication management and HCU among patients receiving chronic treatment with these medications. PENURIE study was a quasi‐experimental before‐and‐after study conducted on the French nationwide health insurance database (*SNDS*).

### 2.2 Ethics

The data treatment of the present feasibility study (PPI‐PENURIE) was registered in conformity with the MR‐004 General Data Protection Regulation principles in the French National Commission for Information Technology and Civil Liberties (*CNIL*) registry under number 23‐053. The study protocol was approved by the French National Institute of Health and Medical Research (*INSERM*) Ethics Committee on 11/09/2023 under number 23‐1022. The patients included in the study were required to sign a commitment form as well as a consent form authorising the use of their data. They also completed a disclosure of conflicts of interest form, in line with the other members of the PENURIE team.

### 2.3 Patient Involvement Method

The patient involvement method used in this study was to set up a Patients Committee alongside the usual Scientific Committee [5]. This aims to reach a high intensity involvement qualified as ‘Partnership’ in the patient engagement continuum [21]. In mirror to the composition of the Scientific Committee, the Patients Committee was to be composed of 5 to 10 members meeting the following criteria [22]: (i) older than 18 years old, (ii) have been on medication for a chronic disease of interest in the pharmacoepidemiology study in for at least 2 years (epilepsy, Parkinson's disease, cardiovascular insufficiency, or bipolar disorders), (iii) in a stable phase of their disease, (iv) volunteer to participate, (v) able to share their experience and present their viewpoint within a group in a neutral posture and, (vi) and had no interest related to the pharmacoepidemiological study.

Members of the Patients Committee were recruited through solicitations to patient associations for the relevant pathologies, the research laboratory's patient partner, as well as the departments at the Lyon hospital and their Partnership and Patient Experience in Health programme (PEPS network). The contact email was accompanied by a plain‐language flyer presenting the pharmacoepidemiology study and its objectives (Supporting Information: S2), as well as an information note. Travel expenses and a gift card were provided for the study participants as explained in the flyer.

### 2.4 Feasibility Study Proceedings

The Patients Committee met in parallel with the PENURIE study's scientific committee at key phases of the research project. The organisation and work programme of the Patient Committee, developed according to the different stages of research with materials and activities to facilitate discussion, is available in Table 1 [5, 23]. The meetings were held in person on dates and times chosen by the participants following a survey, according to the availability of all participants. At each meeting, audio recorded, a member of the research team involved in the PENURIE study was present to facilitate exchanges on methodological aspects and was supported by a social psychologist and a researcher, using the workshops' guides available in Supporting Information: S3. A written summary of the meetings was shared with all participants after each meeting.

**Table 1:** Meeting objectives and associated activities.

	Research phase	Meeting objectives	Activities	Level of involvement
Meeting no.1	Patients' training and introduction to the research project	Introducing research design Presenting the *SNDS* and its content Presenting the PENURIE study	Co‐construction of research steps Live implementation of an emulated *SNDS* through a fictitious patient's care pathway	Information
Meeting no.2	Conceptualisation	Specifying and validating research objectives and outcomes	Case study of a drug shortage at the pharmacy Drawing of the post‐pharmacy care pathway	Collaboration
Meeting no.3	Interpretation of the results	Presenting the study results Interpreting the results	Presentation and discussion of each result	Collaboration
Meeting no.4	Dissemination	Creating a communication support for civil society	Co‐construction of visual abstracts using the main results	Co‐construction

### 2.5 Assessment of the Feasibility and Benefits of Patient Involvement Through the Patient Committee

#### 2.5.1 Feasibility of Patients' Involvement

The feasibility of involving patients was assessed by the number of patients who agreed to participate in the Patients Committee compared with the number of patients approached and expected. A minimum of five patients was defined a priori as the target sample size since no formal recommendations exist regarding patient research committee constitution; we guided our committee on focus group recommendations, which state at least 5 participants per group [24]. We defined the feasibility of involvement in the research process as participation in at least 3 out of 4 meetings and by the number of patients ending their participation prematurely (before the end of the study). In addition, feasibility was also assessed qualitatively through an analysis of group dynamics, examining the cohesion, mutual support, and overall functioning of the Patient Committee.

#### 2.5.2 Benefit of Patients' Involvement

The benefit of patient involvement was described by the number of validations or modifications of outcomes in the PENURIE protocol, the number of new proposals for graphical representation, analysis and interpretation of the results, and the completion of a proposal for a lay communication support. All contributions, whether retained or not, were considered, and no minimum threshold was predefined. These criteria were gathered from the recordings and notes taken by the observer and the meeting facilitator.

#### 2.5.3 Patients' Involvment Process

The evaluation of the patient involvement process was assessed using the McMaster Patient and Public Engagement Evaluation Tool (PPEET) [25], French version, consisting of three questionnaires. The two questionnaires relating to patients, partners and the research team perspective were adapted for the PPI‐PENURIE study, while the research organisation questionnaire was not relevant to the study. These questionnaires were used to assess patient involvement in the project from the perspective of the participating patients and the members of PPI‐PENURIE and PENURIE projects (Supporting Information: S4).

### 2.6 Data Analysis

#### 2.6.1 Quantitative Analysis

Indicators relating to the feasibility and benefits of PPI in the study, as well as responses to the questionnaires, were described using numbers and percentages.

#### 2.6.2 Qualitative Analysis

To assess the feasibility of involving patients with researchers in a collaborative project, two complementary analyses were conducted on the audio recording of the second meeting. The analysis was restricted to this meeting, as it was the only one combining a collaborative level of involvement with a sufficient number of participants: the first meeting was limited to introduction and information sharing, while the third and fourth meetings were conducted in disease‐specific subgroups of one to two participants each. The second meeting, dedicated to reviewing, amending, and finalising the study protocol, also represented a key stage of the research process. A group dynamics analysis [26] was conducted to evaluate interactions between Patient Committee members and the researchers involved in the PENURIE study, as feasibility‐oriented objectives call for an analysis centred on interactional processes rather than content alone [26]. This analysis enabled us to assess the cohesion, mutual support, and overall functioning of the group during the collaborative process, dimensions identified as critical to successful patient involvement [27, 28]. Consensus was defined as situations in which participants either agreed with one another or provided complementary contributions converging toward a shared perspective and was calculated as the proportion of themes reaching consensus out of the total number of coded themes.

In addition, a thematic analysis using a deductive method, based on Bardin [29] and conducted with NVivo software (QSR International) [30], was conducted to identify content that could be directly used to meet the meeting's objectives. Prior to coding, a deductive coding framework was developed, in which main categories were based on the predefined objectives and subcategories were derived from the primary and secondary outcomes of the PENURIE protocol. Two researchers (JR, VV) then independently coded the transcribed audio recording using this framework, classifying each exchange according to whether it fell within the anticipated categories or constituted an unexpected theme. A quantification of the correspondence, expressed in frequency, between encoded exchanges and deductive categories was carried out in order to estimate the proportion of themes falling within the expected framework in relation to unexpected emerging themes. The unit of analysis was defined as a coherent thematic segment, corresponding to a single utterance or sentence conveying a distinct idea. To enhance internal validity, a cross‐analysis was performed independently by two researchers. Disagreements between coders were resolved through discussion and, where consensus could not be reached, a third researcher was consulted. Representative verbatim extracts were selected to illustrate each category.

Together, these two analyses were designed to capture two complementary dimensions of feasibility: the quality of group interactions and dynamics, assessed through group dynamics analysis, and the proportion of patient contributions directly relevant to the study protocol, assessed through thematic analysis.

## 3 Results

### 3.1 Feasibility of Patients' Involvement Through the Patient Committee

#### 3.1.1 Patients' Recruitment

**Figure 1:**
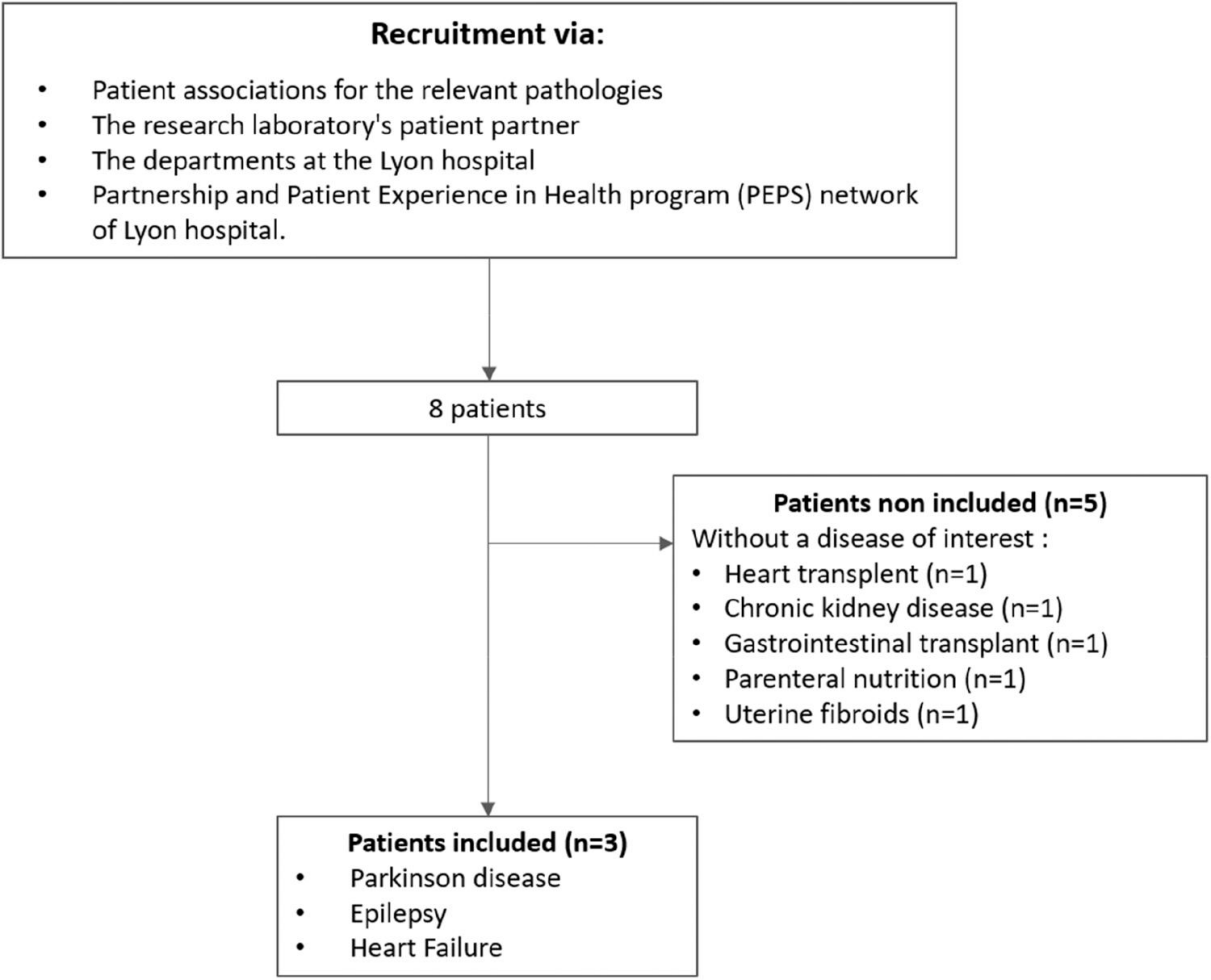
Flow chart of patients' recruitment.

The recruitment was conducted between 1 June 2023 and 31 August 2023. We contacted 10 patient associations, posted the recruitment announcement three times on LinkedIn, and reached out to healthcare professionals and the patient partner, following up with them over a 3‐month period. In total, eight patients contacted the research team: two patients who were members of the patient associations, and six patients from our Hospital Patient Partner network. Among these eight patients, three met the inclusion criteria; the others did not present the targeted conditions. Indeed, the other five patients did not have any of the target conditions (Figure 1). Thus, we included three patients over a 3‐month period, compared with the 5 to 10 patients we had planned to recruit.

#### 3.1.2 Patient Committee Meeting Agenda

**Figure 2:**
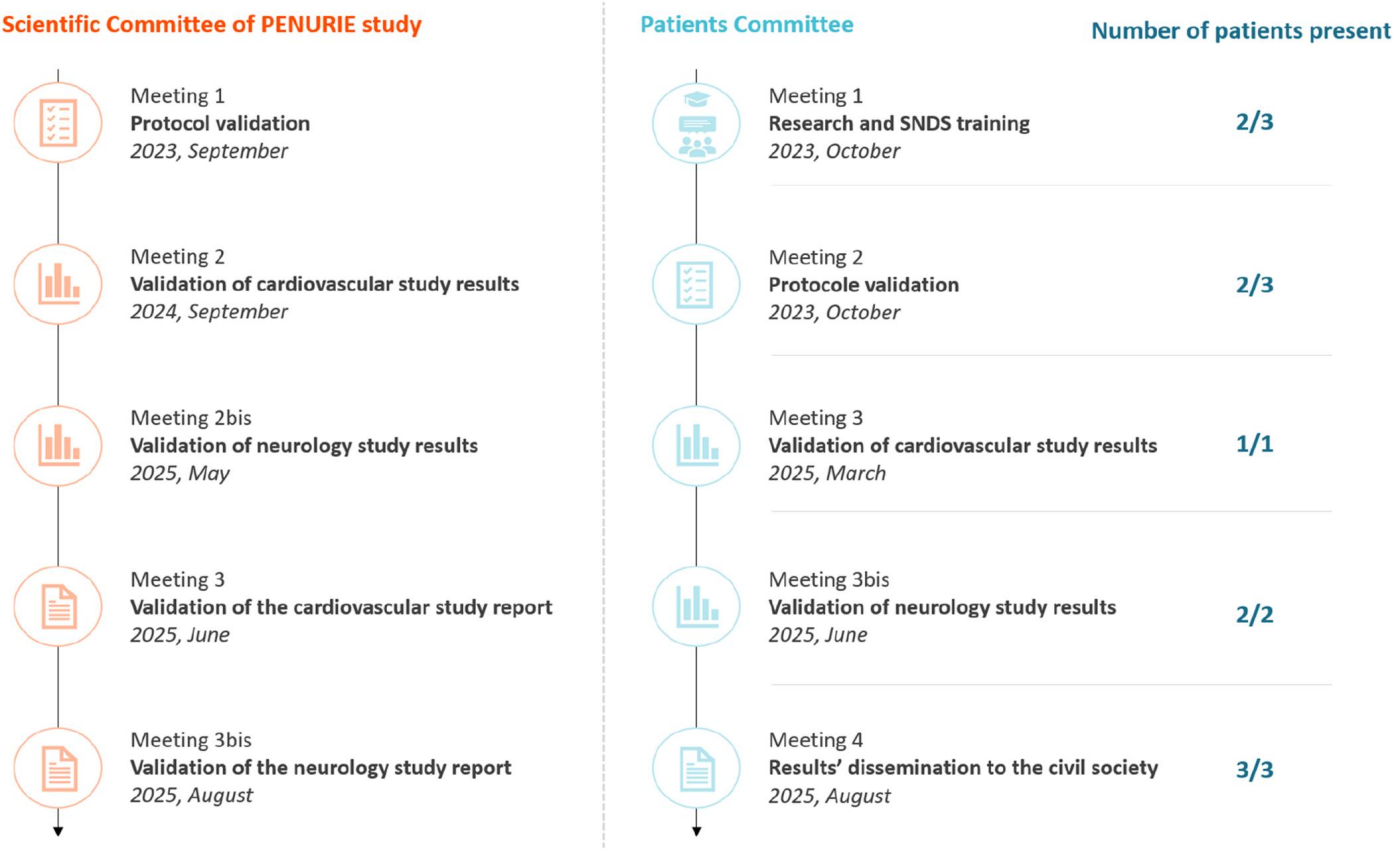
Patient committee meeting agenda.

In total, the patient committee met five times between October 2023 and November 2025, in parallel with the progress of the PENURIE pharmacoepidemiology study (Figure 2). During the results presentation phase, we decided to divide the committee into cardiovascular and neuropsychiatric subgroups, as this facilitated the interpretation of results by allowing participants to focus on conditions with which they were familiar. Attendance at the first two meetings reached two‐thirds of participants. For the meetings dedicated to the results, all patients participated. Finally, at the last meeting, all three patients were present.

#### 3.1.3 Qualitative Findings

**Figure 3:**
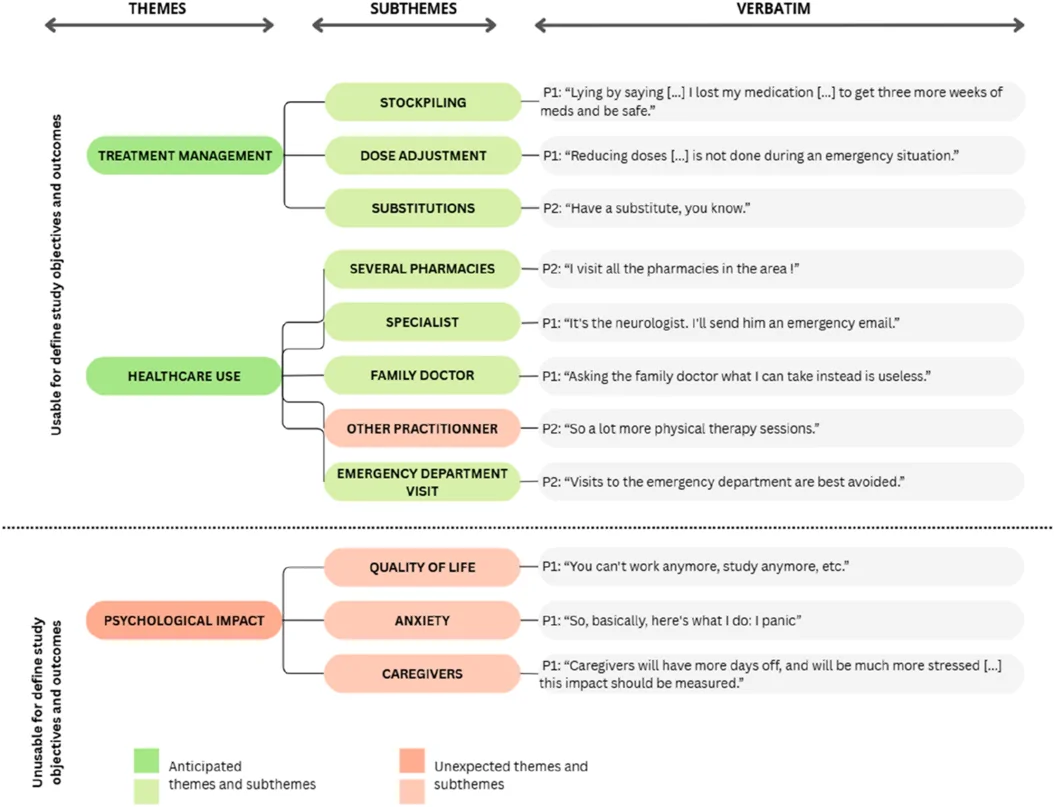
Thematic map of the second meeting*.

Thematic analysis: throughout the meeting, 13% of the exchanges (relative frequency of all discussions) could be classified into the themes related to the study methodology and initially envisaged in the deductive analysis plan (Figure 3). A further 31% corresponded to unexpected themes identified through analysis; although not directly usable for protocol development, these were retained for discussion of the PENURIE study results. The remaining 56%consisted of anecdotes and personal experiences unrelated to the objectives and outcomes of the PENURIE study. In fact, these elements were not linked to their medical history or condition, and consisted of informal discussions (e.g. Marital relationship, opinions on health policy).

Group dynamic analysis: group dynamics analysis demonstrated strong cohesion, as members reached consensus on 83% of themes. Participants often clarified or expanded each other's contributions, and mutual assistance was evident. Most moderator questions focused on technical aspects of *SNDS* data and clarifications of PENURIE outcomes, primarily asked by the patient who was absent from the first *SNDS* and research training session.

*Unusable was defined as when no data were available in the SNDS to construct an indicator related to the stated concept.

### 3.2 Benefit of Patient Involvment in Pharmacoepidemiological Research

All of the proposed amendments made by the patient committee on PENURIE research are summarised in Table 2.

**Table 2:** Summary of proposed amendments by the patients' committee and modifications made by the PENURIE team.

	PENURIE research team	Patient committee proposals	Final elements	Verbatim
Protocol	Three objectives	Three objectives validated	Three initial objectives	P1: ‘Caregivers will have more days off, and will be much more stressed […] this impact should be measured.’
		One added: impact of the shortage on the caregivers' quality of life	Not retained: variable not available in the *SNDS*	
	Thirteen outcomes	Eleven validated	Twelve initial outcomes and the modified one	P2: ‘The loss of comfort will be immediate […] so no more physical therapy sessions, for example.’
		One modified: number of visits to a physical therapist in the health care use criterion		
		One deleted: number of laboratory tests	Not retained: necessary for calculating the total cost	P1: ‘Blood tests are performed regularly, regardless of whether there is a shortage or not; there will be no difference.’
Results	Results presentation	One additional analysis: number of visits to the general practitioner	Analysis conducted by the research team	P3: ‘Before analysing the number of visits to the emergency room, I would look at the family doctor.’
Discussion		Three discussion elements proposed: – psychosocial impact – return to the standard treatment after the switch – utilisation of phone versus in person visite	Three elements retained	P3: ‘Substitution causes anxiety for patients, even if the change only concerns the dosage form.’

#### 3.2.1 Amendments to the Research Protocol

The Patient Committee first validated the three research objectives of the PENURIE study. It also proposed adding an objective to assess the impact of shortages on caregivers' quality of life. This proposal was not accepted by the research team because quality of life is not an existing variable in the *SNDS*. Next, regarding the 13 outcomes proposed by the research team, the Patient Committee validated 11 of them. The Committee proposed modifying one of the outcomes by adding the number of visits to a physical therapist to the healthcare use criteria. This suggestion was accepted by the research team. The criterion for assessing health care costs was not validated by the committee but retained in the protocol by the research team. Patients wanted the reimbursed amounts for laboratory tests to be excluded from the calculation, as any additional test would be part of routine care and, in their view, the shortage would not affect the number of tests performed. However, this proposal was not retained because it is necessary for calculating the total cost, as described in the objectives.

#### 3.2.2 Analysis of Results

After conducting the PENURIE study, two meetings were held in order to present the main results, gather the Patient Committee's analyses of these, and collect their discussion elements. After the presentation of the main results by the statistician of the PENURIE study, the Patient Committee requested an additional analysis concerning the cardiovascular component. The patients wanted a statistical model that was explanatory, not only descriptive, regarding the number of visits to the general practitioner. The proposal was accepted, and this analysis was carried out by the research team.

#### 3.2.3 Discussion Elements

Regarding the discussion, the patient committee highlighted three points; all were retained by the research team and will be added to the PENURIE study article in the discussion section. First, even though it's not measurable in the *SNDS*, the committee wanted the psychosocial impact of shortages to be discussed. Concerning treatments' substitutions, patients were surprised by how many people returned to the standard treatment after a period of substitution. In their view, if a patient tolerates the new treatment, they should remain on it to avoid additional changes. Finally, the patients explained that they would be more likely to contact their specialists by phone to inquire about their options if they had difficulties finding a treatment than visiting face to face.

#### 3.2.4 Results Dissemination

The Patient Committee has produced two Visual Abstracts in plain language with the aim of linking them to scientific publications from the PENURIE study and publishing them within their associative networks.

### 3.3 Evaluation of the Patient Involvement Process

Among the three participants in the Patient Committee, all had a master's degree as their highest level of education. In addition, all had prior involvement in the healthcare sector through patient associations or patient‐partner activities (involvement in hospital departments, therapeutic education services and, in one case, a patient associated with university medical courses).

Patients and project members' responses are presented in the Supporting Information: S5. Members of the Patient Committee appeared satisfied with their participation (3/3 strongly agreed with satisfaction with implication). They felt they had received the necessary information to fulfil their role (3/3 strongly agreed with Question 5), with one member also emphasising the clarity and educational approach of the moderators: ‘The speakers were very educational’. All participants also reported that the research team had taken their differing views into account. To improve the process, one participant suggested increasing the number of participants in future committees: ‘A few more patients to brainstorm in pairs?’. Finally, participants highlighted as a strength of this approach the value of bringing an external patient perspective: ‘The outside and pragmatic perspective’, as well as a good involvement methodology: ‘Methodology [illegible], good involvement of patient partners’.

The project members were also satisfied with the integrity of the process, except with regard to logistical and informational needs. Indeed, the timeframe of the PENURIE study did not allow for the establishment of a meeting schedule or the provision of clear deadlines for participants: *‘*It was difficult to be able to give patients answers about upcoming meeting dates’. Finally, all members expressed interest in receiving training or accessing additional tools to support patient involvement in pharmacoepidemiological research, particularly concerning recruitment and the selection of appropriate methods.

## 4 Discussion

This study is the first French feasibility assessment of a patient involvement approach within a pharmacoepidemiological study using the *SNDS*. Our findings illustrate the added value of integrating patients' experiential knowledge, which contributed to the refinement of one study outcome and identified discussion points about the PENURIE results. However, the feasibility of implementing this approach through a Patient Committee appeared limited. Recruitment challenges, difficulties in convening all participants, and patients' limited familiarity with *SNDS* data collectively constrained their contributions. Moreover, despite good cohesion among committee members, the thematic analysis and observations of group dynamics suggest that moderators faced challenges in leveraging experiential knowledge to achieve the meeting objectives.

Patient recruitment emerged as one of the main factors limiting the feasibility of this approach, a barrier previously identified both in a survey of French academic pharmacoepidemiology researchers on the feasibility of patient involvement [13], and in the scientific literature [17]. Furthermore, members of the project team emphasised the need for access to training or practical tools to facilitate patient recruitment in participative pharmacoepidemiological studies. One of the main factors facilitating recruitment is the support of the research team, either through clinicians, whose relationships with hospital departments facilitate the identification of potential participants [16], or through patient associations, which can help mobilise their members [17, 31]. Another incentive for involvement would be financial compensation for time spent on research activities as well as reimbursement of related expenses [32]. However, the issue of compensation for patients involved remains a subject of debate within the patient and research communities. In the study of R. Dhamanaskar et al., 2024, half of the respondents felt they were not compensated enough, while others, on the contrary, viewed participation as necessarily voluntary [33]. For administrative reasons, we were only able to offer compensation in the form of gift cards, which may not be the most appropriate option [34].

Thematic analysis and group dynamics analysis of the second meeting revealed that only a small proportion of the discussions directly addressed the central objective of the involvement (discussing, refining, and validating the objectives and criteria for evaluating the PENURIE study). The low proportion of protocol‐related exchanges (13%) should therefore be interpreted alongside the positive group dynamics findings: while the relational conditions for successful collaboration were met, the translation of patients' experiential knowledge into protocol‐relevant contributions remained limited. One of the primary explanatory factors was a lack of patients' knowledge of the available data and their understanding of the complex methodologies inherent to developing a study protocol based on medical and administrative databases, as evidenced by participants' own comments and transcript analysis, a barrier consistent with the literature [17, 31, 35]. In 2014, Snape et al. conducted a Delphi study that highlighted a consensus on several barriers, such as the use of overly complex scientific language and patients' lack of experience in research [35]. Thus, the development of dedicated tools or short training courses for stakeholders would be a lever for the feasibility of involving patients in pharmacoepidemiology studies. While our training workshop incorporated an interactive activity (simulation of a real care pathway and a translation step by step into the database), it was certainly not designed to cover the entire subject matter in just 2 h. In addition, the absence of follow‐up materials (such as videos or revision sheets) may have limited knowledge retention and the ability of patient partners to engage more fully with the technical aspects of the protocol. In addition, the researchers themselves had received no prior training on patient involvement and identified access to tools and resources as a key need in the final questionnaire to conduct future involvement processes.

A second explanatory factor was the difficulty experienced by the PENURIE team moderators in mobilising patients' experiential knowledge. Nevertheless, the presence of a trained moderator (psychologist) helped to reduce digressions, an approach recommended in the literature to mitigate this issue [36]. Moreover, each meeting included informal exchange time, which may have contributed to the strong group cohesion [37]. Beyond facilitation, a mismatch between the meeting format and patients' expectations may have limited the expression of experiential knowledge. A facilitator could be specifically trained in eliciting patients' experiential knowledge: Gross and Gagnayre (2017) [38] distinguish implicit experiential knowledge, which remains tacit unless actively elicited, from explicit experiential knowledge, which can be shared and mobilised for collective decisions, a distinction that highlights the facilitation skills needed to turn lived experience into actionable input.

In addition, researchers' implicit assumptions about the nature and value of patient contributions may have influenced the categorisation of exchanges as directly relevant or unrelated to the study objectives, a potential bias inherent to the asymmetrical relationship between researchers and patient partners documented in numerous studies [36, 37, 39]. To address this potential bias, the rigorous cross‐analysis conducted independently by two researchers, with arbitration by a third, aimed to mitigate this risk. This point was also emphasised by Snape et al., with a consensus reached in the first round on the barrier ‘Researchers' attitudes towards relinquishing power and control’ [35]. To address these difficulties, more systematic reporting of stakeholder involvement approaches in scientific publications, as recommended [5], would be valuable. This approach would enable researchers to become familiar with these approaches and, in the long term, would promote collaborations in which experiential knowledge is recognised and legitimised by the scientific community.

This study has several limitations. The small sample size limited the diversity of perspectives collected. Recruitment took place during the summer period, which may have limited uptake. Furthermore, having only one patient representative per condition may have constrained the depth of experiential exchanges, as homogeneous groups sharing the same condition have been shown to foster richer interactions and peer support [40]. Future initiatives should therefore consider recruiting multiple patients per condition, while remaining that larger groups may also affect group dynamics [24]. In addition, all three patient participants had a high level of education, suggesting strong literacy and an understanding of research, and were familiar with health involvement activities. While this facilitated their ability to openly share experiential knowledge and to engage in dissemination work, it may not reflect the broader population of patients who could be involved in such initiatives. Notably, even with this favourable profile (high educational attainment), contributions to protocol refinement remained limited, suggesting that the barriers observed are likely attributable to the inherent complexity of pharmacoepidemiological methods, training was too short, or difficulty in applying experiential knowledge through the proposed activities. Future initiatives involving patients, especially if those patients lack prior research experience and have a more diverse educational background, would benefit from additional training, facilitation and more diverse methods designed to provoke experiential knowledge, such as structured discussion tools or narrative approaches such as the Photovoice method [41, 42]. Furthermore, the patient involvement process was evaluated using a questionnaire one was filled out incorrectly. Thus, our assessment of the participants' views on the barriers and facilitators of patient involvement remains limited. We conducted a thematic analysis using a deductive method, allowing us to evaluate the relevance of themes raised by patients. However, the predefined analytical framework, combined with the structured format of the meeting activities, may have inadvertently constrained the facilitation and driven discussions toward anticipated categories, potentially limiting the space for unanticipated contributions. Finally, our results were context‐dependent and cannot be generalised; however, the detailed description of the study context, participants and methods provided aims to support transferability to similar settings [43].

Despite these limitations, the PPI‐PENURIE study was the first of its kind in France to involve patients in pharmacoepidemiology using medico‐administrative data. In addition, patients remained involved even within the organisational constraints imposed by the PENURIE study's time frame, further supporting the view that they are motivated to participate in such initiatives. Finally, the discussions highlighted elements not captured in the SNDS, such as patients' anxiety or certain behaviours adopted to obtain treatment, demonstrating how experiential knowledge can enrich the interpretation of pharmacoepidemiological findings.

## 5 Conclusion

This study highlighted the potential feasibility under specific conditions of patient involvement in a pharmacoepidemiological research conducted using the *SNDS*. Participants reported overall satisfaction, and benefits of this approach were observed in the PENURIE study, including contributions to protocol modification and proofreading, as well as the identification of key discussion points. However, several barriers were also identified, including well‐known challenges related to participant recruitment as well as a mismatch between patients' availability and the often‐lengthy timelines of research projects.

Additionally, the perceived need for further training has enabled us to develop resources specifically designed for this type of research, which are currently undergoing validation on a larger scale with a view to rolling them out to the French pharmacoepidemiology research community.

## Author Contributions

**Julie Rigoureau:** investigation, writing – original draft, writing – review and editing, visualisation, data curation, formal analysis. **Manon Verroul:** formal analysis, data curation, writing – review and editing. **Valentine Vieillard:** data curation, formal analysis, writing – review and editing. **Anne Termoz:** project administration, writing – review and editing. **Laurie Panse:** writing – review and editing, resources. **Anaïs Havet:** methodology, writing – review and editing. **Marie Viprey:** conceptualisation, writing – review and editing, validation, supervision, methodology. **Julie Haesebaert:** conceptualisation, writing – review and editing, supervision, methodology, validation.

## Ethics Statement

The study was conducted after approval (approval number 23‐1022) from the INSERM Ethical Evaluation Committee (*Institut national de la santé et de la recherche médicale, INSERM*). This study complied with the General Data Protection Regulation and the French regulation while conforming to the reference methodology MR‐004 (number 23‐053 in the general treatment register of Hospices Civils de Lyon). Written informed consent/assent to participate was obtained from each participant in the study.

## Conflicts of Interest

This article represents the views of the authors and does not necessarily represent the opinion of the French Medicines Agency.

## Supporting information


Supporting File

## Data Availability

All data produced in the present study are available upon reasonable request to the corresponding authors.
