# Supplementary material for: Involving Patients in Pharmacoepidemiology Studies Conducted on Healthcare Administrative Database: Results of a Feasibility Study in France
Source: Health Expect. 2026 Sep 21;29(5):e70879. doi: 10.1111/hex.70879 (PMC13591218; doi:10.1111/hex.70879)
Supplement: Supplementary file 1 [file HEX-29-e70879-s001.docx]

# Title

**Involving patients in pharmacoepidemiology studies conducted on healthcare administrative database: results of a feasibility study in France**

# Supplementary file 1: GRIPP2 Long Form

| **Section and topic** | **Item** | **Reported on page No** |
| --- | --- | --- |
| Section 1: Abstract of paper | | |
| 1a: Aim | Report the aim of the study | 1 |
| 1b: Methods | Describe the methods used by which patients and the public were involved | 1 |
| 1c: Results | Report the impacts and outcomes of PPI in the study | 1 |
| 1d: Conclusions | Summarise the main conclusions of the study | 1 |
| 1e: Key words | Include PPI, “patient and public involvement,” or alternative terms as keywords | 2 |
| Section 2: Background to the paper | | |
| 2a: Definition | Report the definition of PPI used in the study and how it links to comparable studies | 2 |
| 2b: Theoretical underpinnings | Report the theoretical rationale and any theoretical influences relating to PPI in the study | 2 |
| 2c: Concepts and theory development | Report any conceptual models or influences used in the study | NA |
| Section 3: Aims of paper | | |
| 3: Aim | Report the aim of the study | 3 |
| Section 4: Methods of paper | | |
| 4a: Design | Provide a clear description of methods by which patients and the public were involved | 3 |
| 4b: People involved | Provide a description of patients, carers, and the public involved with the PPI activity in the study | 4 |
| 4c: Stages of involvement | Report on how PPI is used at different stages of the study | 4-5 |
| 4d: Level or nature of involvement | Report the level or nature of PPI used at various stages of the study | 5 |
| Section 5: Capture or measurement of PPI impact | | |
| 5a: Qualitative evidence of impact | If applicable, report the methods used to qualitatively explore the impact of PPI in the study | 5-6 |
| 5b: Quantitative evidence of impact | If applicable, report the methods used to quantitatively measure or assess the impact of PPI | 5-6 |
| 5c: Robustness of measure | If applicable, report the rigour of the method used to capture or measure the impact of PPI | NA |
| Section 6: Economic assessment | | |
| 6: Economic assessment | If applicable, report the method used for an economic assessment of PPI | NA |
| Section 7: Study results | | |
| 7a: Outcomes of PPI | Report the results of PPI in the study, including both positive and negative outcomes | 8 |
| 7b: Impacts of PPI | Report the positive and negative impacts that PPI has had on the research, the individuals involved (including patients and researchers), and wider impacts | 9 |
| 7c: Context of PPI | Report the influence of any contextual factors that enabled or hindered the process or impact of PPI | 7 |
| 7d: Process of PPI | Report the influence of any process factors, that enabled or hindered the impact of PPI | 7 |
| 7ei: Theory development | Report any conceptual or theoretical development in PPI that have emerged | NA |
| 7eii: Theory development | Report testing of theoretical models, if any | NA |
| 7f: Measurement | If applicable, report all aspects of instrument development and testing (eg, validity, reliability, feasibility, acceptability, responsiveness, interpretability, appropriateness, precision) |  |
| 7g: Economic assessment | Report any information on the costs or benefit of PPI | NA |
| Section 8: Discussion and conclusions | | |
| 8a: Outcomes | Comment on how PPI influenced the study overall. Describe positive and negative effects | 12 |
| 8b: Impacts | Comment on the different impacts of PPI identified in this study and how they contribute to new knowledge | 12 |
| 8c: Definition | Comment on the definition of PPI used (reported in the Background section) and whether or not you would suggest any changes | NA |
| 8d: Theoretical underpinnings | Comment on any way your study adds to the theoretical development of PPI | 12 |
| 8e: Context | Comment on how context factors influenced PPI in the study | 12 |
| 8f: Process | Comment on how process factors influenced PPI in the study | 12 |
| 8g: Measurement and capture of PPI impact | If applicable, comment on how well PPI impact was evaluated or measured in the study | 12 |
| 08h: Economic assessment | If applicable, discuss any aspects of the economic cost or benefit of PPI, particularly any suggestions for future economic modelling. | NA |
| 8i: Reflections/critical perspective | Comment critically on the study, reflecting on the things that went well and those that did not, so that others can learn from this study | 13 |

# Supplementary file 2: Recruitment flyer

# Supplementary file 3: Meetings’ guide

**Meeting n°1 – Guide**

**► SET UP**

Facilitated by:

- **Moderator:** Julie Rigoureau
- **Observer:** Manon Verroul

**► INTRODUCTION OF THE TWO FACILITORS AND PARTICIPANTS**

**► ICE-BREAKER ACTIVITY: PHOTO-EXPRESSION**

Objective: To allow each patient to introduce themselves through a playful activity and to foster group dynamics.

Instructions: A set of photographs will be laid out on a table. Each participant will be invited to choose a photo that evokes the theme “Chronic disease.” Once everyone has selected a photo, a round of introductions will take place. Each participant will introduce themselves by giving their first name, their condition (if they wish), and explaining why they chose that photo.

Time allocated: 30 minutes

Materials: Photo-expression set “chronic disease”

Probing questions: What common themes did you all express?

**► PRESENTATION OF IMPULSE TEAM, THE PENURIE STUDY, AND PARTICIPANTS’ ROLE**

*"To begin, I will explain present the research team briefly and the PENURIE study."*

| Sequence | Objective | Materials |
| --- | --- | --- |
| Presentation of research agenda | Understand the framework in which these meetings take place | Power Point |
| General presentation of IMPULSE team | Discover the research team’s affiliations and the institutional context of PENURIE study | Power Point |

Time allocated: 30 minutes

Materials: Projector, computer, slides

Probing questions: Which aspects would you like us to explore further?

**► INTRODUCTION TO THE SNDS**

*"I will now briefly present the key stages of a research protocol and the SNDS. All the PENURIE study is conducted on the SNDS"*

| Sequence | Objective | Materials |
| --- | --- | --- |
| Diagram of the PENURIE context and *SNDS* use | Understand the study context (primary objectives) and data used | Power Point |
| Research protocol diagram | Understand the main steps of a research protocol | Power Point |
| Presentation of the data available in the *SNDS* | Gain a broad understanding of the data underlying the PENURIE study | Power Point |

Time allocated: 60 minutes

Materials: Projector, computer, slides

Probing questions:

- Which points would you like us to revisit?
- At which stages of a research protocol do you think you could contribute?
- Which important information do you think the PENURIE study might miss?

**Meeting n°2 – Guide**

**► SET UP**

Facilitated by:

- **Moderator:** Manon Verroul / Julie Rigoureau
- **Observer:** Antoine Ardillon

Materials: Computer, projector, whiteboard/paperboard, colored markers

**► FEEDBACK ON THE PREVIOUS MEETING**

*Are there any points from the meeting summary we sent you that you would like clarified?*

**Time allocated:** 10 minutes

**► IMPACT OF DRUG SHORTAGES ON MEDICATION MANAGEMENT**

Facilitation: Julie Rigoureau, Manon Verroul

Discussion / questions: Antoine Ardillon

Materials: A4 sheets, whiteboard, magnets, markers

Objective: To allow participants to describe behaviors they would adopt in the event of a drug shortage, with the ultimate goal of defining the objectives and outcome measures of the PENURIE study.

**Scenario instruction:** *"You go to the pharmacy to collect your treatment. Upon arrival, you are informed that it is unavailable due to a drug shortage."*

Participants respond orally; responses are recorded by the observer.

| Predefined Theme | Activity | Questions | Probing questions |
| --- | --- | --- | --- |
| Impact of drug shortages on medication management | Scenario | What would you do? | What other reactions might you have in this situation?  You did not mention “X behavior”—could this be a possible reaction? Why? |

Predefined subthmes: stockpiling, dose adjustment, substitutions

**► IMPACT OF DRUG SHORTAGES ON THE CARE PATHWAY**

Facilitation: Julie Rigoureau, Manon Verroul
Discussion / questions: Antoine Ardillon

Materials: A4 sheets, whiteboard, magnets, markers

Objective: To encourage participants to imagine their care pathway following a drug shortage, in order to define secondary objectives and related outcome measures.

**Scenario instruction:** *"Starting from the reference point ‘drug shortage,’ how would you describe the evolution of your care pathway and your disease?*

Participants respond orally; responses are recorded on a timeline.

| Predefined theme | Activity | Questions | Probing questions |
| --- | --- | --- | --- |
| Impact of drug shortages on care pathway | Timeline | From this point, representing drug shortage, how do you think your disease evolve and how your care pathway could be modified? | Imagine you have no access to your treatment for XXX—what might happen?  What do you think your healthcare utilization would be? |

Predefinde subthmes: several pharmacies, visits to general practitionner and/or specialist and/or another professional, visits to emergency department

**Meeting n°3 – Guide**

**► SET UP**

Facilitated by:

- **Moderator:** Julie Rigoureau / Hugo Marthinet

Materials: Computer, projector, whiteboard/paperboard, colored markers

**► PRESENTATION OF THE RESULTS**

The statistician from the PENURIE study will present the various results.

Participants will be invited to reflect on:

- The presentation of results (figures, tables)
- The relevance of the results in relation to their care pathways

**Time allocated:** 2 hours

**Materials:** Projector, computer, slides (or others depending on the workshop)

| **How do you interpret this result?** | **Does this result resonate with your care pathway? Why?** | **How do these results reflect patient care pathways?** | **What do you think of its presentation? Would you prefer another format?** |
| --- | --- | --- | --- |
| **Result 1** |  |  |  |
| **Result X** |  |  |  |

**Meeting n°4 – Guide**

**► SET UP**

Facilitated by:

- **Moderator:** Julie Rigoureau / Manon Verroul

**► DISSEMINATION OF RESULTS**

Objective: To develop a communication support tailored to the civil society.

**Time allocated:** 2 hours

Materials: Computer, projector, whiteboard/paperboard, colored markers, Canva visual abstract template, printed key results

**► QUESTIONNAIRE**

Each patient completes the end-of-study questionnaire.

**Time allocated:** 10 minutes

# Supplementary file 4: McMaster questionnaires

**End-of-study Questionnaire PPI-PENURIE**

**Project section**

*The Public and Patient Engagement Evaluation Tool has been licensed under a Creative Commons Attribution-NonCommercial-Share Alike 4.0 International License. ©2018, Julia Abelson and the PPEET Research-Practice Collaborative. McMaster University. All rights reserved.*

**Part A. Integrity of Design and Process**

1. The perspectives of those who will be most affected by the outputs of this project were reflected through those who participated in the engagement.

|  | Strongly disagree |  | Disagree |  | Neither agree nor disagree |  | Agree |  | Strongly agree |
| --- | --- | --- | --- | --- | --- | --- | --- | --- | --- |

2. The financial, logistical and information needs of participants (e.g., travel, dietary, interpretive, childcare, etc.) were accommodated.

|  | Strongly disagree |  | Disagree |  | Neither agree nor disagree |  | Agree |  | Strongly agree |
| --- | --- | --- | --- | --- | --- | --- | --- | --- | --- |

3. Adequate time was allocated to plan and implement the engagement component.

|  | Strongly disagree |  | Disagree |  | Neither agree nor disagree |  | Agree |  | Strongly agree |
| --- | --- | --- | --- | --- | --- | --- | --- | --- | --- |

4. The goals for the engagement component were shared with participants.

|  | Strongly disagree |  | Disagree |  | Neither agree nor disagree |  | Agree |  | Strongly agree |
| --- | --- | --- | --- | --- | --- | --- | --- | --- | --- |

5. Participants were told how the input from the engagement component would be used

by the organization.

|  | Strongly disagree |  | Disagree |  | Neither agree nor disagree |  | Agree |  | Strongly agree |
| --- | --- | --- | --- | --- | --- | --- | --- | --- | --- |

**Part B. Influence and Impact**

6. Was a summary report of the engagement component of the project prepared?

|  | Yes |
| --- | --- |
|  | No 🡪 Skip to part C (10) |

7. Who was the report shared with? (Please select all that apply)

|  | Individuals who participated in the engagement activity |
| --- | --- |
|  | Management within your organization |
|  | Board members |
|  | Partners |
|  | Others, please specify: |

8. Please select from the following list how the engagement input was shared with relevant individuals and/or groups within or outside the organization.

|  | Written reports were made available to interested individuals |
| --- | --- |
|  | Presentations, briefings to individuals or groups |
|  | Informal communications strategies (e.g., emails, phone conversations) |
|  | Other strategies were used. Please describe these below: |
|  | We did not share the engagement input 🡪 Why was the input not shared with others? |

**Part C. Collaboration, Common Purpose and Partnerships**

9. We worked with other organizations as part of the engagement component of this activity.

|  | No 🡪 Skip to Part D (Q13) |
| --- | --- |
|  | Yes 🡪 Please list the organizations you worked with below. |

10. We were able to identify shared goals with our partners through this process.

|  | Strongly disagree |  | Disagree |  | Neither agree nor disagree |  | Agree |  | Strongly agree |
| --- | --- | --- | --- | --- | --- | --- | --- | --- | --- |

11. Do you plan to collaborate with these partners again in the future?

|  | No |
| --- | --- |
|  | Yes 🡪 Please describe your plans below. |

**Part D. Final Reflections**

12. Overall, I was satisfied with the engagement component of this project.

|  | Strongly disagree |  | Disagree |  | Neither agree nor disagree |  | Agree |  | Strongly agree |
| --- | --- | --- | --- | --- | --- | --- | --- | --- | --- |

13. The engagement component added value to the project it supported.

|  | Strongly disagree |  | Disagree |  | Neither agree nor disagree |  | Agree |  | Strongly agree |
| --- | --- | --- | --- | --- | --- | --- | --- | --- | --- |

14. As a result of my involvement in the engagement component associated with this project, I will be comfortable leading future engagement activities.

|  | Strongly disagree |  | Disagree |  | Neither agree nor disagree |  | Agree |  | Strongly agree |
| --- | --- | --- | --- | --- | --- | --- | --- | --- | --- |

15. I would like to participate in PPE training to build my capacity to do more of this work.

|  | No |
| --- | --- |
|  | Yes 🡪 what PPE-related skills would you be interested in developing? |

# Supplementary file 5: Answers to the questionnaires

**Participant section**

| **N°** | **Question** | **Answer** |
| --- | --- | --- |
| **Part A: Communication and assistance for participation** | | |
| 3 | *I had a clear understanding of the PENURIE study objective* | 2/3 Strongly agree  1/3 Agree |
| 4 | *I have the logistical support I need to participate (transports, childcare, etc.) in the PENURIE study* | 2/3 Strongly agree  1/3 Neither agree nor disagree |
| 5 | *I have enough information to fulfill my role within the study* | 3/3 Strongly agree |
| 6 | *Would you like to say anything else about how your involvment in the PENURIE study was facilitated?* | “The speakers were very educational” |
| **PartB: Sharing your views** | | |
| 7 | *I was able to express my views freely* | 3/3 Strongly agree |
| 8 | *I feel that my views were heard* | 3/3 Strongly agree |
| 9 | *The workshop participants represented a wide range of viewpoints* | 2/3 Strongly agree  1/3 Agree |
| 10 | *Would you like to add anything about how you share your opinions?* | “The listening made sharing easier.” |
| **Part C: Impact and influence of patient involvement** | | |
| 11 | *I think that the Patients’ involvement in the PENURIE study achieved its objectives* | 2/3 Strongly agree  1/3 Agree |
| 12 | *I am confident the input provided through this initiative will be used by the PENURIE team* | 2/3 Strongly agree  1/3 Agree |
| 13 | *I think the input provided through this activity will make a difference to the work of the research team* | 1/3 Strongly agree  1/3 Agree  1/3 Neither agree nor disagree |
| 14 | *What else would you like to tell us about the impact of your participation in the Patient Committee of the PPI-PENURIE project?* | “The perspective of civil society allows us to adapt the presentation of results to make them accessible to as many people as possible.”  “A few more patients to brainstorm in pairs?” |
| **Part D: Final thoughts** | | |
| 15 | *Thanks to my participation in the PENURIE study, I feel better informed about the impact that drug shortages can have on patients with chronic diseases* | 3/3 Strongly agree |
| 16 | *Overall, I was satisfied with this engagement initiative* | 3/3 Strongly agree |
| 17 | *My involvement in the PENURIE study was a good use of my time* | 2/3 Strongly agree  1/3 Agree |
| 18 | *What were the strengths of patients’ involvement in the PENURIE study?* | “Methodology [illegible], good involvement of patient partners”  “The outside and pragmatic perspective”  “Allow patients to take a step back from media reports or prejudices circulating among patients.” |
| 19 | *What could be improved about the patients’ involvement in the PENURIE study?* | “Contact the association's communications manager to make an official announcement”  « [illegible] » |
| 20 | *What else would you like us to know about your experience with patient involvement?* | “Not often enough in my opinion in research, but it's getting better! Thank you” |

**Project section**

| **N°** | **Question** | **Réponses** |
| --- | --- | --- |
| **Part A: Integrity of Design and Process ntégrité de la conception et du processus** | | |
| 1 | *The perspectives of those who will be most affected by the outputs of this project were reflected through those who participated in the engagement.* | 3/3 Agree |
| 2 | *The financial, logistical and information needs of participants (e.g., travel, dietary, interpretive, childcare, etc.) were accommodated.* | 1/3 Disagree  1/3 Agree  1/3 Strongly agree  “It was difficult to be able to give patients answers about upcoming meeting dates” |
| 3 | *Adequate time was allocated to plan and implement the engagement component.* | 3/3 Strongly agree |
| 4 | *The goals for the engagement component were shared with participants.* | 3/3 Strongly agree |
| 5 | *Participants were told how the input from the engagement component would be used*  *by the organization.* | 3/3 Strongly agree |
| **Part B: Influence et impact** | | |
| 6 | *Was a summary report of the engagement component of the project prepared?* | 3/3 YES |
| 7 | *Who was the report shared with? (Please select all that apply)* | Epi-Phare (research team partners) |
| 8 | *Please select from the following list how the engagement input was shared with relevant individuals and/or groups within or outside the organization* | Written reports were made available to interested individuals (EPI-PHARE) |
| **Part C: Collaboration, Common Purpose and Partnerships** | | |
| 9 | *We worked with other organizations as part of the engagement component of this activity* | 3/3 NO |
| **Part D: Final Reflections** | | |
| 12 | *Overall, I was satisfied with the engagement component of this project* | 1/3 Neither agree nor disagree  2/3 Agree |
| 13 | *The engagement component added value to the project it supported* | 2/3 Agree  1/3 Strongly agree |
| 14 | *As a result of my involvement in the engagement component associated with this project, I will be comfortable leading future engagement activities* | 1/3 Neither agree nor disagree  1/3 Agree  1/3 Strongly agree |
| 15 | *I would like to participate in PPI training to build my capacity to do more of this work.* | 3/3 YES  “Find ways to facilitate the recruitment and mobilization of patients/the public in similar initiatives, identify the most appropriate communication channels for getting information to the patients/public concerned.”  “Better understand the drivers of each method/activity to better leverage experiential knowledge”. |
|  | | |
